# Perceptions and Degree of Satisfaction with the Health Sciences University Educational Community Regarding the Measures Adopted for the Prevention of COVID-19 in the Academic Year 2020/2021

**DOI:** 10.3390/ejihpe11030076

**Published:** 2021-09-06

**Authors:** Óscar Rodríguez-Nogueira, Raquel Leirós-Rodríguez, Enedina Quiroga-Sánchez, Mª José Álvarez-Álvarez, Lorena Álvarez-Barrio

**Affiliations:** 1 SALBIS Research Group, Nursing and Physical Therapy Department, Universidad de León, 24401 Ponferrada, Spain; orodn@unileon.es (Ó.R.-N.); equis@unileon.es (E.Q.-S.); mjalva@unileon.es (M.J.Á.-Á.); 2 Nursing and Physical Therapy Department, Universidad de León, 24401 Ponferrada, Spain; lalvb@unileon.es

**Keywords:** teaching, higher education, Coronavirus infections, pandemics, health sciences, security measures, protocols

## Abstract

The COVID-19 pandemic caused the start of the academic year 2020/2021 to be conditioned by health and safety regulations. The present research was defined with the aim of analyzing the degree of satisfaction and perceptions on the establishment of bubble groups and pairs and on the use of audiovisual platforms for the development of theoretical and practical university teaching in three degrees of health sciences. A cross-sectional descriptive study was carried out on a representative sample of students and teachers of health sciences in Ponferrada (*n* = 285). Specific questionnaires designed for this study were completed virtually during April and May 2021. The results indicate that that satisfaction was moderate–high. The perception of the influence of bubble pairs on the quality of teaching can be interpreted as very low. These results increase with the age and academic and professional experience of students and faculty members, respectively. However, the participants belonging to physiotherapy considered that the quality of teaching had worsened much more compared to their counterparts in nursing and podiatry.

## 1. Introduction

The COVID-19 pandemic caused the start of the academic year 2020/2021 to be conditioned by health and safety regulations [1]. This special situation of public health emergency has led to multiple changes in the living and working habits of millions of people [2]. From a contextual point of view, there has been a profound social, economic and value change that has affected society as a whole, especially organizations, institutions and teachers [3]. Specifically, Spanish university students have had to continue attending online classes, and their social life has been limited by the prohibition to go outside [4].

However, this health crisis can be interpreted as an experience from which to learn to help the educational community per se and, especially, to prepare for a new similar situation, which must go beyond the use of more Information and Communication Technology (ICT) and more digital and virtual tools [5]. In other words, the new context has caused a turning point for teachers at all educational levels, forcing them to observe what is happening in the classroom, analyze the reasons for certain actions and plan new and better didactic proposals, among other types of consequences [6]. In the pandemic crisis, university students, despite being exposed to the stressors of the reduction in academic relationships, as well as restrictive measures, have used their digital skills to enhance their professional training, finding in them an outstanding resource for their personal growth. E-learning has represented a fundamental tool through which to continue with the formative curricula, facilitating the emotional well-being of young students. The affinity of e-learning has had a buffering effect on the worsening of quality of life implied by the restrictive measures [7]. In fact, Tang et al. [8] detected that the psychological consequences of COVID-19 could be severe. Furthermore, they suggested that psychological interventions that reduce fear and improve sleep duration should be made available to college students who have stayed at home and that priority should be given to graduating students and those from the most affected areas.

Recently, governmental, health and educational institutions had to face, at the beginning of the academic year 2020/2021, the implementation of protocols and action plans for the prevention of COVID-19. Due to the administrative organization of autonomous communities in which competencies in education have been transferred, such protocols are different in each autonomous community [9]. The measures contemplated are varied and diverse; however, in general, all autonomous communities have agreed on the need to reduce the number of students per classroom (down to a maximum of 20 students), the implementation of blended learning (especially in the university environment), the development of support and monitoring plans, special cleaning and distancing measures and the delegation of greater responsibility to centers and teachers [10].

In the specific case of university teaching, some centers have established bubble groups; that is, subgroups within the student body that would always share a classroom, respecting the ratio of students per square meter available. At the same time, for the development of practical teaching in pairs, bubble pairs have been established. In other words, pairs of students have been defined, who would share experiences and learning throughout all the practical classes of the entire course [11]. Owing to the advantages of digital tools, such as the fact that commuting is no longer needed, allowing social distance and flexibility in time, they have been maintained in this academic year, combined with face-to-face teaching, to facilitate the delivery of virtual teaching by teachers and/or the virtual attendance of students to theoretical classes [12]. This distance modality is of special interest in those cases of home isolation for being COVID-19 positive or for being in direct contact with someone who has tested positive, having to keep the quarantine at home to preserve the health of other people [13]. 

For all these reasons, the present research was defined with the aim of analyzing the degree of satisfaction and perceptions on the establishment of bubble groups and pairs and on the use of audiovisual platforms for the development of theoretical and practical university teaching in three degrees of health sciences (nursing, physiotherapy and podiatry). This study was based on the previous hypothesis that satisfaction had been moderate and that, although the methodologies used to date were preferable, theoretical and practical teaching had been adapted to the circumstances. To verify this, a descriptive cross-sectional study was carried out on a representative sample of students and teachers of health sciences in Ponferrada (University of León, Spain).

## 2. Materials and Methods

### 2.1. Experimental Design and Sample

A cross-sectional descriptive study was carried out on a representative sample of students and teachers of health sciences in Ponferrada (University of León, Spain). The census of this educational center in the academic year 2020/2021 was 455 students and 54 teachers. According to these data, considering a sample heterogeneity of 50%, a margin of error of 5% and a confidence level of 95%, it would be necessary to reach a sample size of 220 individuals. A sample of 285 participants was obtained for this study, with a response and participation rate of 56%.

### 2.2. Procedure

Specific questionnaires designed for this study were completed virtually during April and May 2021. An email with the protocol and instructions was sent to all faculty and students of the Faculty of Health Sciences. From this mailing, we obtained a sample of 285 people who expressed their desire to participate in the study on a voluntary basis. It was explained to all participants that the study respected the principles of the Declaration of Helsinki (rev. 2013) and the Data Protection Act 15/1999. In addition, all of them had to sign the informed consent for participation in this research. The research protocol was approved by the Ethics Committee of the University of León (code: ÉTICA-ULE-027-2021).

### 2.3. Research Instrument and Variables Analyzed

The instrument used was an ad hoc questionnaire designed to collect information on the perception of students and teachers of the anti-COVID-19 standards implemented in the Faculty of Health Sciences of Ponferrada as a result of the Action Plan for the adaptation of teaching to health requirements. The questionnaire was elaborated taking into account other works on the subject and was validated by means of expert judgment [14,15]. To this end, we requested the help of three women and two men who specialized in education and health. A first draft of the questionnaire was designed and sent to the aforementioned group, and they sent their comments and suggestions. With this information, the questionnaire was corrected and modified and sent again so that they could assess the suitability of the questions in the corrected instrument. Finally, these experts gave their assessment, showing a level of agreement of 88%; thus, we considered the questionnaire to be valid for application to the 285 participants, according to the criteria of Polit et al. [16]. Reliability was determined by standardizing the survey administration protocol for all participants.

The questionnaire consisted of a total of seven items aimed at ascertaining the perception and opinions of students and teachers on the measures imposed:

- “In relation to the creation of bubble couples for the development of practical teaching, do you consider that it has influenced the quality of teaching?” with five Likert-type response options (1 indicated that it had not influenced at all and 5 that it had totally influenced).
- “If you consider that the creation of bubble couples has changed the quality of teaching, in what way has it done so?” with five Likert-type response options (1 indicated that it had worsened it and 5 that it had improved it).
- “Could you give the reason for the previous answer?” qualitative open-ended question.
- “In relation to teaching in theoretical sessions and seminars, do you think that theoretical skills and knowledge have been adequately developed?” with five Likert-type response options (1 indicated that they had not been developed at all and 5 that they had been fully developed).
- “In relation to teaching during practical sessions, do you think that practical competencies and skills have been developed adequately?” with five Likert-type response options (1 indicated that they had not been developed at all and 5 that they had been fully developed).
- “Have you taught or been taught telematically via video call due to being in confinement?” Question with dichotomous response option: “Yes” or “No”.
- “If you have attended or taught while in confinement, what has been your degree of satisfaction with this modality?” with five Likert-type response options (1 indicated that the degree of satisfaction had been null and 5 that the perceived satisfaction had been very high).

Finally, the following sociodemographic variables were included: gender, age, years of association with the faculty and the degree of association (nursing, physiotherapy or podiatry).

### 2.4. Statistical Analysis

For the analysis of the results, sample subgroups were defined according to age: G1, with participants aged 18 to 25 years (*n* = 247); G2, with participants aged 26 to 35 years (*n* = 18); G3, with participants aged 36 to 45 years (*n* = 15); and G4, with participants older than 46 years (*n* = 8).

Descriptive measures (frequencies, percentages, mean and standard deviation) were used to characterize the sample. The chi-square ratio test and Cramer’s V statistic were used to test the equality of proportions of the groups in a large database according to the different groups of age, teaching experience and tenure.

The *t*-tests and Cohen’s d statistic were used to determine differences between sexes and the ANOVA test and the partial eta-squared statistic were used to analyze the questions of the questionnaire with a continuous response option (from 0 to 5), according to the degree of affiliation.

All statistical analyses were performed with Stata version 12 (StataCorp., College Station, TX, USA), and statistical significance was always established at a value of *p* < 0.05.

## 3. Results

The sample consisted of 285 participants, of whom 202 (70.9%) were women (Table 1). No statistically significant differences were found between the sexes in the age variable or in the type of relationship with the faculty. However, differences were found in the years of association with the center and in the degree of origin.

The first item (“In relation to the creation of bubble couples for the development of practical teaching, do you consider that it has influenced the quality of teaching?”) showed a result of 3.2 ± 1.4 points out of 5. The results of this item did not vary significantly when analyzed individually by sex, degree or type of relationship with the center (*p* > 0.05). However, the obtained responses did correlate inversely with the variable age (r = −0.2; *p* = 0.02) and with the years of association with the center (r = −0.2; *p* = 0.002).

The second item (“If you consider that the creation of bubble couples has modified the quality of teaching, in what way it has done so?”) showed a result of 2.9 ± 1.2 points out of 5. The results of this item did not vary significantly when analyzed individually by sex or type of relationship with the center (*p* > 0.05). However, the obtained responses were statistically different depending on the degree (*p* < 0.0001; η^2^_p_ = 0.1): the scores obtained by the physiotherapy students (2.6 ± 1.1 points) were significantly different from those of the nursing (3.2 ± 1.1 points) and podiatry students (3.6 ± 1.2 points). The obtained scores did not correlate with the variable age (*p* > 0.05) but did correlate with the years of association with the center (r = −0.2; *p* = 0.03).

**Figure 1:**
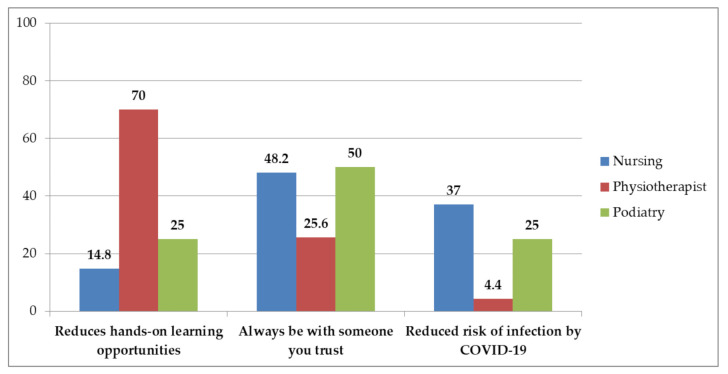
Responses to “Why does the establishment of bubble couples change the quality of education?” based on degree.

In Item 3, which was an open-ended qualitative response (“Could you give the reason for the previous answer?”), the most frequent responses (54.3%) were identified as those related to the fact that it reduces the possibilities of practical learning. Another two large groups of responses were also identified, which were related to the fact that it enhances learning by ensuring coexistence with a trusted partner with whom there is a good relationship (in 32.6% of cases) and that it provides peace of mind, as it allows performing tasks that involve contact with the lowest risk of COVID-19 contagion (13.2%). This proportion of the identified responses did not differ between sexes, the type of relationship with the center or age (*p* > 0.05). On the other hand, differences were identified according to the degree (X^2^ = 36.38; *p* < 0.0001; V = 0.38). Thus, for the participants belonging to physiotherapy, the main reason transmitted was, in a negative sense, due to the reduction of practical learning possibilities implied by always being with the same partner (70% of the members of this subgroup). On the other hand, the reason most frequently given by the nursing and podiatry participants was positively formulated as the possibility of always being with a partner they trust and with whom they have a good relationship (Figure 1).

In relation to the items that evaluated the acquisition of competencies and knowledge, the evaluation of the theoretical and seminar contents was 4.2 ± 1 points. The results of this item did not vary significantly when analyzed by sex, degree or type of relationship with the center (*p* > 0.05). The obtained scores correlated with the variable age (r = 0.2; *p* = 0.03) but not with the years of association with the center (*p* > 0.05). The assessment of the acquisition of skills and knowledge related to the practical classes was evaluated with a mean of 4.3 ± 0.1 points. The results on this item did not vary significantly when analyzed by sex or type of relationship with the center (*p* > 0.05). However, the obtained results did differ between the analyzed degrees (*p* = 0.0001): the rating given by the nursing students was statistically lower (4 ± 1.2 points) than that given by the physiotherapy students (4.5 ± 0.8 points). The obtained scores did not correlate with the variable age or with the years of association with the center (*p* > 0.05).

The item “Have you taught or been taught telematically via video call due to being in confinement?” showed that 39% of the sample used this platform. The analysis of the obtained responses did not differ according to sex, degree or age subgroups (*p* > 0.05). However, it did differ according to the link with the center (X^2^ = 4.01; *p* = 0.04; V = 0.11): 60% of the teachers have taught classes telematically compared to 37.4% of students who have been taught by the same means.

In relation to the degree of satisfaction with this teaching modality, the result was 3.2 ± 1.4 points out of 5. In addition, the results of this item did not vary significantly in the individualized analysis by sex or type of link with the center (*p* > 0.05). However, they did as a function of degree (*p* = 0.004; η^2^_p_: 0.08): the podiatry participants showed significantly lower scores (2.4 ± 1.3 points) compared to the nursing (3.5 ± 1.4 points) and physiotherapy participants (3.3 ± 1.3 points). The obtained responses were not correlated with the variable age (*p* > 0.05), but they correlated with the years of association with the center (r = 0.2; *p* = 0.03).

## 4. Discussion

The aim of this study was to analyze the degree of satisfaction and perceptions about the establishment of bubble groups and pairs for the development of theoretical and practical university teaching in three degrees of health sciences (nursing, physiotherapy and podiatry). In light of the obtained results, it can be affirmed that satisfaction has been moderate–high.

The perception of the influence of bubble pairs on the quality of teaching can be interpreted as very low. This was similar among the different careers and between students and teachers; that is, regardless of the area of knowledge and position as a participant in the teaching–learning activity, the establishment of this measure does not seem to influence it. In particular, it has been identified that the older the age, the lower the perception of influence within the students and teachers themselves. It is important to highlight that user perceptions and satisfaction are some of the significant indicators of service quality [17,18]. However, this research identified that, in more advanced courses (or as the teaching experience of the faculty members increases), the evaluation of the characteristics in which teaching is carried out is minimized, and the teaching itself is more highly valued. This contradicts previous studies that indicate that the capacity to adapt to new scenarios decreases with age due to physical and mental exhaustion, which appears more prematurely than in previous decades [19,20].

However, the participants belonging to physiotherapy considered that the quality of teaching had worsened much more compared to their counterparts in nursing and podiatry, who expressed an average in their responses much closer to a neutral perception (close to three; that is, they thought that the quality of teaching had neither worsened nor improved). This may be due to the particularities in the academic organization of this degree. That is to say, in the degree of physiotherapy, the teaching load associated with practical classes is much higher than in the other degree programs included in this study [21,22,23]. In fact, this possible explanation is consistent with what was identified in the qualitative item included in the survey. Specifically, seven out of ten participants belonging to physiotherapy expressed, as the main reason, a negative response due to the reduction of practical learning possibilities implied by always being with the same partner. On the other hand, the reason most frequently given by the nursing and podiatry participants was positively formulated as the possibility of always being with a partner they trust and with whom they have a good relationship. Nevertheless, in the physiotherapy subgroup, the evaluation of the acquisition of skills and practical content was very positive. Thus, it could be interpreted that, despite the recognition of the limitations in learning implied by the new dynamics implemented, the fundamental teaching objectives have been satisfactorily achieved by the participants. 

In the current academic year, more than one-third of the sample of this study have used at some point a telecommunications platform (specifically Google Meet) to give or receive online teaching. This platform has been one of the most used tools during the COVID-19 confinement in 2020 [24]. It has been mostly used by the participating faculty members to teach the theoretical classes compared to the students who have received such online teaching. Therefore, it can be assumed that there have been more confinements due to illness or contact with COVID-19 among the faculty than among the students, or due to specific personal circumstances of the former group, such as having underage children who have been placed in quarantine (and thus having to take care of them).

Regarding the degree of satisfaction of the participants with the use of the audiovisual telecommunications platform in teaching, it was found to be moderate, with the nursing students expressing a higher degree of satisfaction, followed by the physiotherapy students; on the other hand, the podiatry students expressed a lower degree of satisfaction. Previous studies carried out in circumstances of home isolation obtained a similar or even higher degree of satisfaction than in the present investigation, as they perceived ease of use due to its simple operation and the advantages offered by this platform [12,25]. However, it should be borne in mind that face-to-face teaching improves interpersonal communication, facilitating the establishment of group dynamics. This type of teaching, where students interact with the teacher, can provide greater attendance and follow-up of classes by breaking with the monotony of lectures [26].

This study has methodological limitations that should be recognized. Firstly, the collection of information through this questionnaire with closed and limited response options may bias the participants’ assessments. Secondly, it was not possible to use other instruments to triangulate the results and thus verify the validity of the opinions expressed through the different questions. However, this research presents strengths, such as the timeliness of the moment in which it was carried out, the representativeness of the sample size achieved, the inclusion of all the degrees taught in the participating Faculty of Health Sciences in which the research was developed and the inclusion and comparison of the contributions of students and teachers.

As a future line of research, a contrast analysis is suggested as health safety regulations are modified, and these participants advance in their academic and professional careers.

## 5. Conclusions

In light of the obtained results, it can be affirmed that satisfaction with the implementation of the bubble groups and pairs was moderate–high. These results increase with the age and academic and professional experience of students and faculty members, respectively. In addition, these implemented measures have especially affected practical teaching in the degree of physiotherapy, a degree in which the proportion of practical content is higher and experiential experimentation with peers is of special importance for the proper integration of content.

Consequently, it is necessary to continue working so that academic health protocols for face-to-face classes are carefully planned following national and international guidelines, in order to ensure that students and teachers are safe or at least mitigate the effects of COVID-19, promoting consensus among faculty, students and health and educational institutions.

## Figures and Tables

**Table 1:** Descriptive statistics of the sample.

	All (*n* = 285)	Men (*n* = 83)	Women (*n* = 202)
Age (mean ± standard deviation)	22.7 ± 6.9	22.3 ± 6.7	22.9 ± 7
Type of linkage (*n* (percentage)):			
Teacher	20 (7%)	5 (6)	15 (7.4)
Student	265 (93%)	78 (94)	187 (92.6)
Years of linkage (*n* (percentage)): *			
One	79 (27.7%)	15 (18)	64 (31.7)
Two	114 (40)	34 (41)	80 (39.6)
Three	43 (15.1)	21 (25.3)	22 (10.9)
Four	36 (12.6)	11 (13.3)	25 (12.3)
Between five and nine	3 (1.1)	1 (1.2)	2 (1)
Ten or more	10 (3.5)	1 (1.2)	9 (4.5)
Degree (*n* (percentage)): **			
Nursing	94 (33)	17 (20.5)	77 (38.1)
Physiotherapy	154 (54)	53 (63.8)	101 (50)
Podiatry	37 (13)	13 (15.7)	24 (11.9)

Contrast analysis results X^2^: * *p* < 0.05; ** *p* < 0.01.

## Data Availability

The data presented in this study are available on request from the corresponding author.
